# Evaluating treatments with topical anaesthetic and buccal meloxicam for pain and inflammation caused by amputation dehorning of calves

**DOI:** 10.1371/journal.pone.0198808

**Published:** 2018-06-13

**Authors:** Dominique Van der Saag, Sabrina Lomax, Peter Andrew Windsor, Casey Taylor, Peter John White

**Affiliations:** 1 The University of Sydney, Faculty of Science, Sydney School of Veterinary Science, Sydney, New South Wales, Australia; 2 The University of Sydney, Faculty of Science, School of Life and Environmental Sciences, Sydney, New South Wales, Australia; University of Bari, ITALY

## Abstract

To assess the effects of a topical anaesthetic (TA) and buccal meloxicam (BM) on behaviour, maximum wound temperature and wound morphology following amputation dehorning of beef calves, 50 unweaned Hereford calves were randomly allocated to: (1) sham dehorning / control (CON, n = 14); (2) amputation dehorning (D, n = 12); (3) amputation dehorning with pre-operative buccal meloxicam (DBM, n = 12); and (4) amputation dehorning with post-operative topical anaesthetic (DTA, n = 12). Videos of the calves were captured for 3 h following treatment. Each calf was later observed for 5 min every hour and the frequency and duration of specific behaviours displayed during these focal periods was recorded. Infrared and digital photographs of dehorning wounds were collected from all dehorned calves on days 1, 3 and 7 following treatment. Infrared photographs were used to identify the maximum temperature within the wound area. Digital photographs were used to score wounds based on visual signs of inflammation and healing, using a numerical rating scale of 1 to 3, with morphological aspects of inflammation increasing and morphological aspects of healing decreasing with progressive scores. CON calves displayed fewer head shakes than all dehorned calves at 2 and 3 h following treatment (*P* = 0.025). CON and DTA calves displayed less head turns than DBM calves at 2 h following treatment (*P* = 0.036). CON calves displayed fewer combined point behaviours than all dehorned calves at 2 h following treatment (*P* = 0.037). All dehorning wounds had a greater maximum temperature on days 3 and 7 compared to day 1 (*P* = 0.003). All wound morphology scores decreased from day 1 to day 3 and wound morphology scores of DBM and DTA calves increased from day 3 to day 7 (*P* = 0.03). Although flystrike may have confounded these observations, no clear effects of TA or BM on behaviour, maximum wound temperature or wound morphology following dehorning of calves were observed. Further research is required to evaluate the analgesic efficacy of these products for amputation dehorning of calves.

## Introduction

It is well recognised that amputation dehorning of cattle causes pain and distress [1–6]. Despite this, it remains a common procedure as it reduces the risk of injury to cattle and people, damage to infrastructure, space requirements for housing and transport, and the incidence of both hide damage and bruised carcasses at slaughter [7].

The Australian animal welfare guidelines for cattle state that surgical procedures should be performed with pain relief [8]. However, additional time, skills and expense required for administration of injected forms of anaesthesia and analgesia are major barriers to uptake by the majority of producers [9]. These constraints are a particular issue for cattle industries that are large and extensive and where polled animals are rare. Such conditions are typical of northern Australian beef production systems, where horned Brahman cattle dominate, herd numbers are large and animals are raised and managed under very extensive pastoral settings [10]. In these systems, calves are ‘marked’ (mustered and subjected to husbandry management procedures including ear tagging, ear notching, branding, castration and dehorning) only once or twice a year, resulting in dehorning of large numbers of animals of various ages [9].

In recent years, the practical limitations to using anaesthesia and analgesia during surgical husbandry procedures in livestock have been addressed through the development and registration of ‘farmer applied’ products. A topical anaesthetic (TA) gel, Tri-Solfen^®^ (Bayer Animal Health, NSW, Australia), originally developed for spray-on application to open wounds induced during mulesing of lambs, has recently been registered for tail docking of lambs and surgical castration of lambs and calves [11, 12]. This TA has also been shown to provide post-operative anaesthesia of dehorning wounds comparable to that provided by a cornual nerve block of lignocaine [13]. In addition to TA, a gel containing the non-steroidal anti-inflammatory drug (NSAID) meloxicam, Ilium^®^ Buccalgesic OTM (Troy Laboratories Ltd Pty, NSW, Australia), was developed for administration into the buccal cavity for oral trans-mucosal delivery. A previous study using buccal meloxicam (BM) demonstrated a reduction in pain-related behaviours following surgical castration and tail docking in lambs [14].

The aim of this study was to investigate the effects of TA and BM on behaviour, maximum wound temperature and wound morphology following amputation dehorning in beef calves. These measures were assessed as indications of pain and inflammation. We predicted that TA and BM would reduce pain-related behaviours and that BM would reduce maximum wound temperature and morphological aspects of wound inflammation.

## Materials and methods

### Animals and treatments

The experiment was approved by the Animal Ethics Committee of the University of Sydney (Approval No. 5832). Fifty unweaned Hereford beef calves (6 to 8 months old) were sourced from a commercial property on the southern tablelands of NSW, Australia. Of the 50 calves, 21 were steers and 29 were heifers. Steers had been castrated at 3 to 4 months of age. All castration wounds were fully healed by the time this study commenced.

Calves were blocked by sex and randomly allocated to one of four treatment groups by use of computer generated random numbers using Microsoft Excel 2010 (Microsoft Office Excel 2010, version 15.0.4569.1506): (1) sham dehorned / control (CON, *n* = 14); (2) dehorned (D) (D, *n* = 12); (3) dehorned with pre-operative administration of buccal meloxicam (Ilium^®^ Buccalgesic OTM; Troy Laboratories, NSW, Australia) (DBM, *n* = 12); and (4) dehorned with post-operative application of topical anaesthetic (Tri-Solfen^®^; Bayer Animal Health Australia, NSW, Australia) (DTA, *n* = 12).

Upon inspection at the point of randomisation and treatment, six calves were identified as being polled. These calves were allocated to the control treatment group that did not require dehorning and a polled (P) or horned (H) phenotype was noted for each CON animal.

Dehorning was performed by a single, experienced technician using a yearling cup dehorner, (Dominion Yearling Cup; Bainbridge Pty Ltd, Qld, Australia) designed for use on cattle up to 18 months of age. Dehorning was performed by placing the open cup over the horn, applying downward pressure and closing the handles in a scissor-like action to excise the horn and immediate surrounding tissue. Sham dehorning was performed by placing the open cup over the horn and applying light downward pressure without closing the handles so that no physical injury occurred.

The TA contained lignocaine (40.6 g / L), bupivacaine (4.2 g / L), cetrimide (5g / L) and adrenaline (24.8 mg / L) in a gel formulation. For DTA calves, 4 mL of product was applied by spraying each wound immediately post dehorning, covering the entire wound and immediate surrounding skin. The product was applied using a household spray bottle and the amount applied per spray (2 mL) was calibrated by spraying the TA into a cup and using a 3 mL syringe to measure the volume.

The BM contained meloxicam (10 mg / mL) in a gel formulation. For DBM calves, 1 mL / 20 kg body weight of BM was administered into the buccal cavity between the dorsal molar teeth and the buccal mucosa of the oral cavity, using a drench-like gun applicator, delivering meloxicam at a dose rate of 0.5 mg / kg body weight.

### Experimental design

Before and during the experiment, calves and their mothers were held in a paddock adjacent to the cattle handling facilities. Both cows and calves had *ad libitum* access to water and pasture. The trial was conducted across 8 days in summer, with data collected on days 0, 1, 3 and 7 following treatment. Calf handling required that the calves were drafted from their mothers into one of three smaller holding yards adjacent to the cattle race, then drafted through the race and restrained in a cattle crush (Ultimate Cattle Crush; RPM Australia-Pacific Pty Ltd, Qld, Australia) and a headbail (Superlock Headbail; RPM Australia-Pacific Pty Ltd, Qld, Australia) for treatment on day 0 and for data collection on days 1, 3 and 7. The calves were then released into the paddock with their mothers following data collections. On day 0, the calves were processed through the race twice. At the initial draft, the calves were ear tagged, weighed and spray painted with an identification number (1 to 50) on both sides and the back of the body, with buccal meloxicam administered to DBM calves 25 min prior to dehorning. At the second draft, calves were dehorned or sham dehorned and DTA calves were treated with TA. On days 1, 3 and 7 of the trial, the calves were processed through the race once for data collection and all wounds were sprayed with spinosad (Extinosad Aerosol for Wounds; Elanco Animal Health, NSW, Australia) for fly control.

### Observations and measurements

#### Behaviour

On day 0, the calves were observed immediately following treatment for 3 h of behavioural observations. This was conducted in two round yards (80 m^2^ each) adjacent to the cattle handling facilities with three video cameras (HD 1080p Sports Action Cam; Sony Australia Ltd, NSW, Australia), attached at various points along the fence of each yard to capture videos of the cattle from all angles of the yards. The videos continuously recorded the frequency or duration of certain specified behaviours displayed by each calf. For analysis, 5-minute focal periods were examined every hour for 3 h following treatment. The frequency or duration of behaviours were recorded using an observational data software package, The Observer^®^ XT 12 (Noldus Information Technology, Gelderland, The Netherlands). An ethogram was designed using this software whereby behaviours were categorised as states or points (Table 1). The ethogram was derived from previously published studies on dehorning [4, 5]. State behaviours were quantified by duration (s) and point behaviours were quantified by frequency.

**Table 1:** Ethogram developed for behavioural observations conducted on calves following treatment.

Behaviour	Description
**States**^1^	
Walk	Walking forwards or backwards in any style at any pace.
Stand	Standing in any style.
Lie	Lying down completely on the ground in any style.
Head down	Holding head below brisket.
Scratch	Raising a hind leg and scratching part of the body or scratching body against the yard fence.
Lick	Turning head back and licking body with lips or tongue, or both.
**Points**^2^	
Head shake	Rapid shaking of the head around a rostral to caudal axis.
Head turn	Rapid turning of the head to either side of the body.
Head paw	Lifting of hind leg and contacting with the head.
Head rub	Rubbing head against another calf or the yard fence.
Ear flick	Rapid movement of one or both ears.

^1^ States are behaviours with measurable duration and are quantified by duration of time (s).

^2^ Points are behaviours without measurable duration and are quantified by frequency.

#### Maximum wound temperature

**Fig 1:**
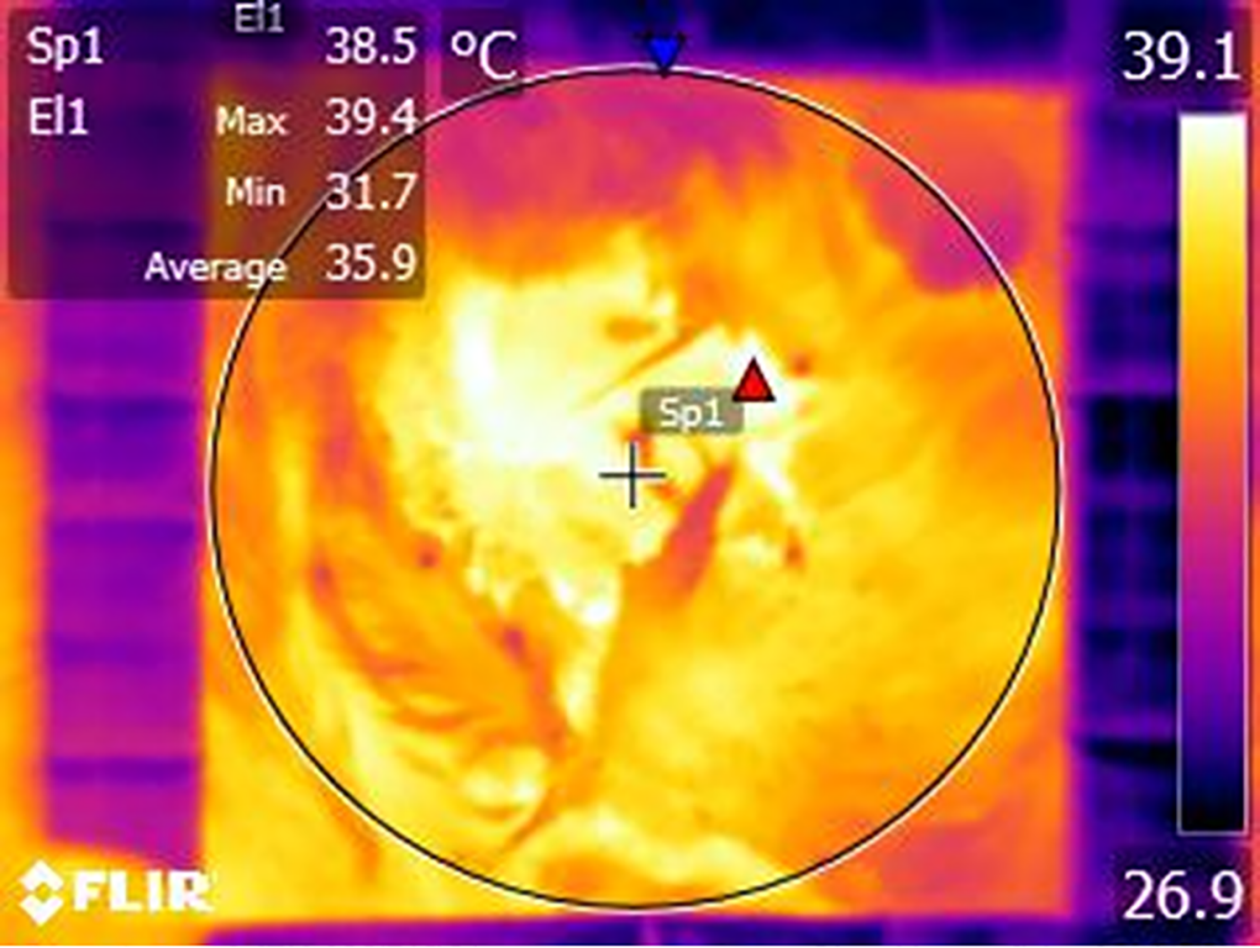
Infrared image of a dehorning wound analysed for maximum surface temperature. A thermal imaging software program, FLIR^®^ Tools Software (FLIR^®^ Systems, Inc., International) was used to calculate maximum surface temperature within a circle drawn inside a cardboard frame which was held over each wound for each photograph.

Infrared photographs of both the left and right wounds were captured from all dehorned calves on days 1, 3 and 7 of the trial using a handheld infrared camera (FLIR^®^E50; FLIR^®^ Systems, Inc., International), with a thermal range of -20°C to 120°C and a sensitivity of 0.045°C. A 10 cm x 10 cm cardboard frame was used to standardise the image area for each photograph. The camera frame was aligned with the cardboard frame held over the wound for each photograph, ensuring the camera lens was at a consistent distance of 0.5 m from the wound for each photograph. This distance, along with an emissivity value of 0.95 were entered into the infrared camera for calibration. Ambient temperature and humidity were monitored and recorded at the time each photograph was captured and were entered into the infrared camera for calibration every 30 min during the data collection period. Images were analysed for maximum temperature using a thermal imaging software program, FLIR^®^ Tools Software (FLIR^®^ Systems, Inc., International). This software allowed for analysis of a specific area using a geometric figure drawn on the photograph. A circle was drawn around the wound within the cardboard frame in each photograph and the maximum temperature within this area was calculated (Fig 1).

#### Wound morphology

**Fig 2:**
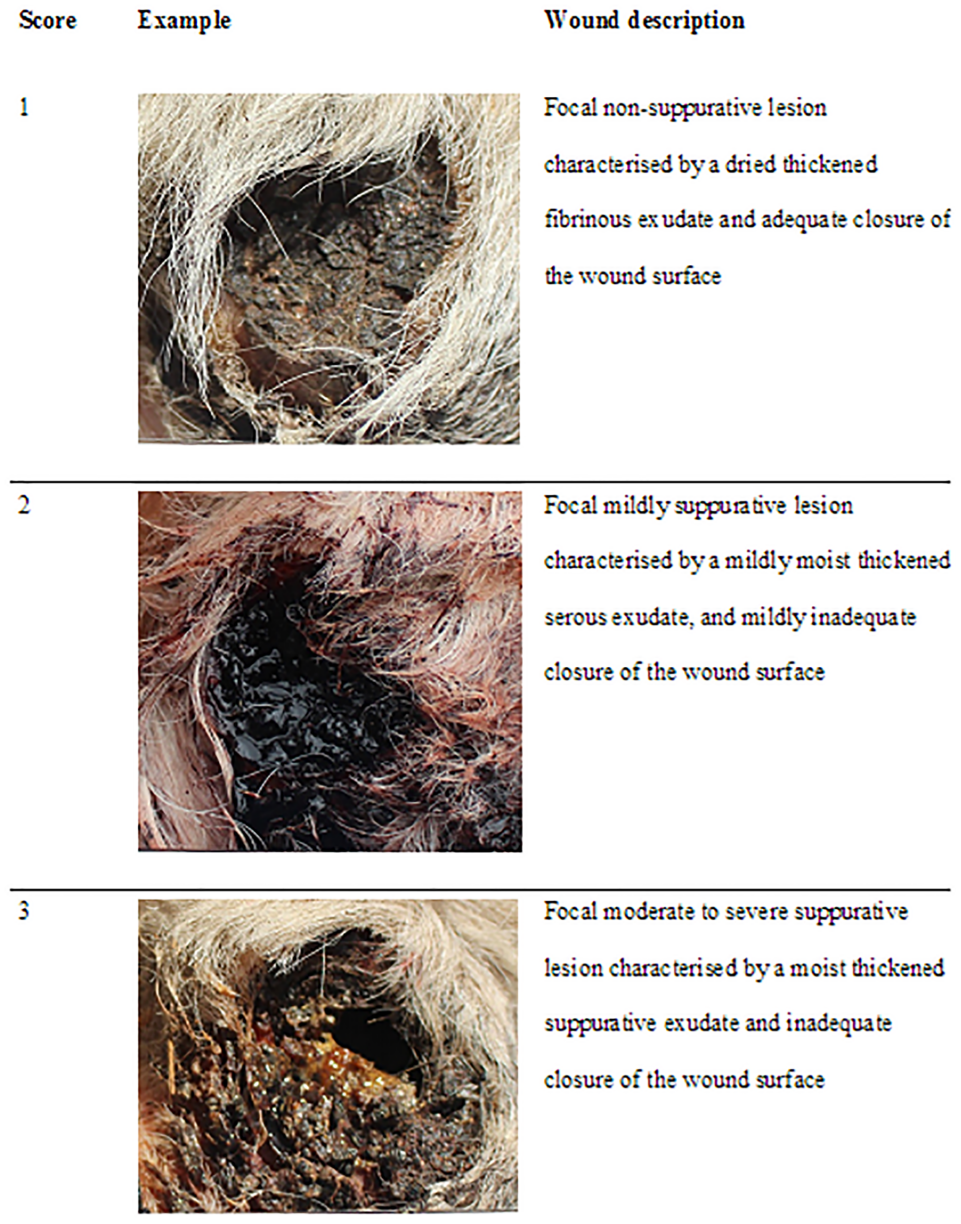
Customised numerical rating scale used to score wound morphology.

Digital photographs of the wound were taken from all dehorned calves on days 1, 3 and 7. These photographs were later scored for morphological aspects of inflammation and healing using a customised numerical rating scale of 1 to 3 (Fig 2).

### Statistical analysis

Data on each state behaviour and maximum wound temperature were subjected to restricted maximum likelihood (REML) for repeated measures using the linear mixed models procedure of Genstat^®^ 17^th^ Edition statistical software (VSN International Ltd, Hertfordshire, UK). Data on each state behaviour displayed by CON calves only was also analysed this way to analyse the effect of a polled phenotype. Data on each point behaviour were subjected to REML for repeated measures using the generalised linear mixed models (GLMM) procedure of Genstat^®^ with a poisson distribution. Data on the combined frequency of all point behaviours was also analysed this way. Data on each point behaviour displayed by CON calves only was also analysed this way to analyse the effect of a polled phenotype. Wound appearance scores were subjected to ordinal logistic regression (OLR) in ASReml^®^ 3.0 statistical software (VSN International, Hertfordshire, UK). For each behaviour displayed by CON calves only, the fixed effect of the model was phenotype (P, H). For each behaviour and combined point behaviours (Table 1), the fixed effects of the model were treatment (CON, D, DBM, DTA) x time-point (h) (1, 2, 3). Data on ambient temperature and ambient humidity was subjected to a Spearman’s rank correlation using the nonparametric correlations procedure of Genstat^®^ to identify a possible collinear effect and determine inclusion of factors in the model for maximum wound temperature. For maximum wound temperature, the fixed effects of the model were treatment (CON, D, DBM, DTA) x day (1, 3, 7) + ambient temperature (variate). Data on ambient temperature and maximum wound temperature was subjected to a Spearman’s rank correlation using the nonparametric correlations procedure of Genstat^®^. For wound appearance, the fixed effects of the model were treatment x day. The random effect for all models was calf ID. Data from the REML analyses are presented as predicted means. Data from the OLR analysis are presented as cumulative odds ratios with the statistical probabilities of wounds displaying scores of *Y* = 1, 2 and 3. For all statistical calculations, *P* values ≤ 0.05 were considered statistically significant.

## Results

### Animals and environment

Calves weighed 235.67 ± 44.83 kg. Average ambient temperatures during the data collection period on days 1, 3 and 7 were 33.89°C, 30.30°C and 33.85°C, respectively. Average ambient humidities during the data collection period on days 1, 3 and 7 were 33.97%, 33.99% and 9.63%, respectively.

### Behaviour

There were 16 missing focal periods due to calves being unidentified in the video footage. Of these missing samples, there were 2 from time-point 1 (2 x CON calves), 5 from time-point 2 (2 x CON, 2 x DBM and 1 x DTA calves) and 9 from time-point 3 (4 x CON, 2 x DBM and 3 x DTA calves). Behaviours influenced by time only are neither presented nor discussed.

There was no significant effect of the polled phenotype on any behaviours displayed by CON calves (*P* > 0.05).

There was a significant treatment x time interaction on the frequency of head shakes (*P* = 0.025) head turns (*P* = 0.036) and combined point behaviours (*P* = 0.037) (Table 2). CON calves displayed fewer head shakes than all dehorned calves at 2 and 3 h following treatment and CON and DTA calves displayed fewer head turns than DBM calves at 2 h following treatment. CON calves displayed fewer combined point behaviours than all dehorned calves at 2 h following treatment. There was no significant effect of treatment on any other behaviours (*P* > 0.05).

**Table 2:** Mean frequency of head shakes, head turns and combined point behaviours displayed by calves in each treatment group within a 5-minute focal sample at each time-point.

Behaviour	*P*–value	Time-point (h)	Mean frequency (± s.e.m.)			
			CON	D	DBM	DTA
Head shakes	0.025	1	0.94^A^^a^ ± 0.42	1.59^A^^a^ 0.60	0.98^A^^a^ ± 0.41	1.80^A^^a^ ± 0.66
		2	0.13^B^^a^ ± 0.13	1.72^A^^b^ ± 0.64	2.24^A^^b^ ± 0.82	2.13^A^^b^ ± 0.76
		3	0.17^B^^a^ ± 0.16	2.76^A^^b^ ± 0.92	0.99^A^^b^ ± 0.43	2.14^A^^b^ ± 0.82
Head turns	0.036	1	2.33^A^^a^ ± 0.51	4.17^A^^a^ ± 0.67	4.17^A^^a^ ± 0.67	4.25^A^^a^ ± 0.68
		2	1.25^A^^a^ ± 0.37	2.08^B^^a^^b^ ± 0.48	3.70^A^^b^ ± 0.70	1.27^B^^a^ ± 0.39
		3	2.10^A^^a^ ± 0.52	1.58^B^^a^ ± 0.42	1.60^B^^a^ ± 0.46	2.22^A^^b^^a^ ± 0.57
Combined point behaviours	0.037	1	1.70^A^^a^ ± 1.14	2.04^A^^a^ ± 1.42	1.87^A^^b^^a^ ± 1.26	2.01^A^^a^ ± 1.39
		2	0.88^B^^a^ ± 0.69	1.58^A^^b^ ± 1.05	2.08^A^^b^ ± 1.58	1.68^A^^b^ ± 1.15
		3	1.32^A^^b^^a^ ± 0.98	2.05^A^^a^ ± 1.43	1.54^B^^a^ ± 1.10	2.08^A^^a^ ± 1.62

CON = sham dehorned / control; D = dehorned; DBM = dehorned with pre-operative buccal meloxicam; DTA = dehorned with post-operative topical anaesthetic.

^a, b^ Values within a row with different superscripts differ significantly at *P* ≤ 0.05.

^A, B^ Values within a column with different superscripts differ significantly at *P* ≤ 0.05.

Descriptive statistics are based on predicted means (*±* s.e.m.).

### Maximum wound temperature

On day 1, infrared photographs from 15 calves (5 x D, 5 x DBM and 5 x DTA calves) were missing due to a temporary technical malfunction with the infrared camera. On day 3, infrared photographs from 1 D calf were missing as they could not be located.

A strong negative correlation (R = -0.89) between ambient temperature and ambient humidity was identified, therefore only ambient temperature was included in the model.

There was a significant effect of day (*P* = 0.003), with greater maximum wound temperatures on days 3 (40.43 ± 0.35 °C) and 7 (40.30 ± 0.40 °C) compared to day 1 (38.83 ± 0.42 °C). There was a significant effect of ambient temperature (*P* < 0.001). A moderate positive relationship between ambient temperature and maximum wound temperature was identified (R = 0.52). There was no significant effect of treatment (*P* = 0.797).

### Wound morphology

Photographs from two calves (1 x D and 1 x DTA calf) on day 3 were excluded due to poor quality. On day 3, it was noted anecdotally that some of the open wounds were in the early stages of flystrike, as indicated by putrefactive odour and weeping of the wound. On day 7, the severity of flystrike had increased, as indicated by the presence of maggots in some open wound sinuses, serous exudate and the characteristic foul odour.

**Fig 3:**
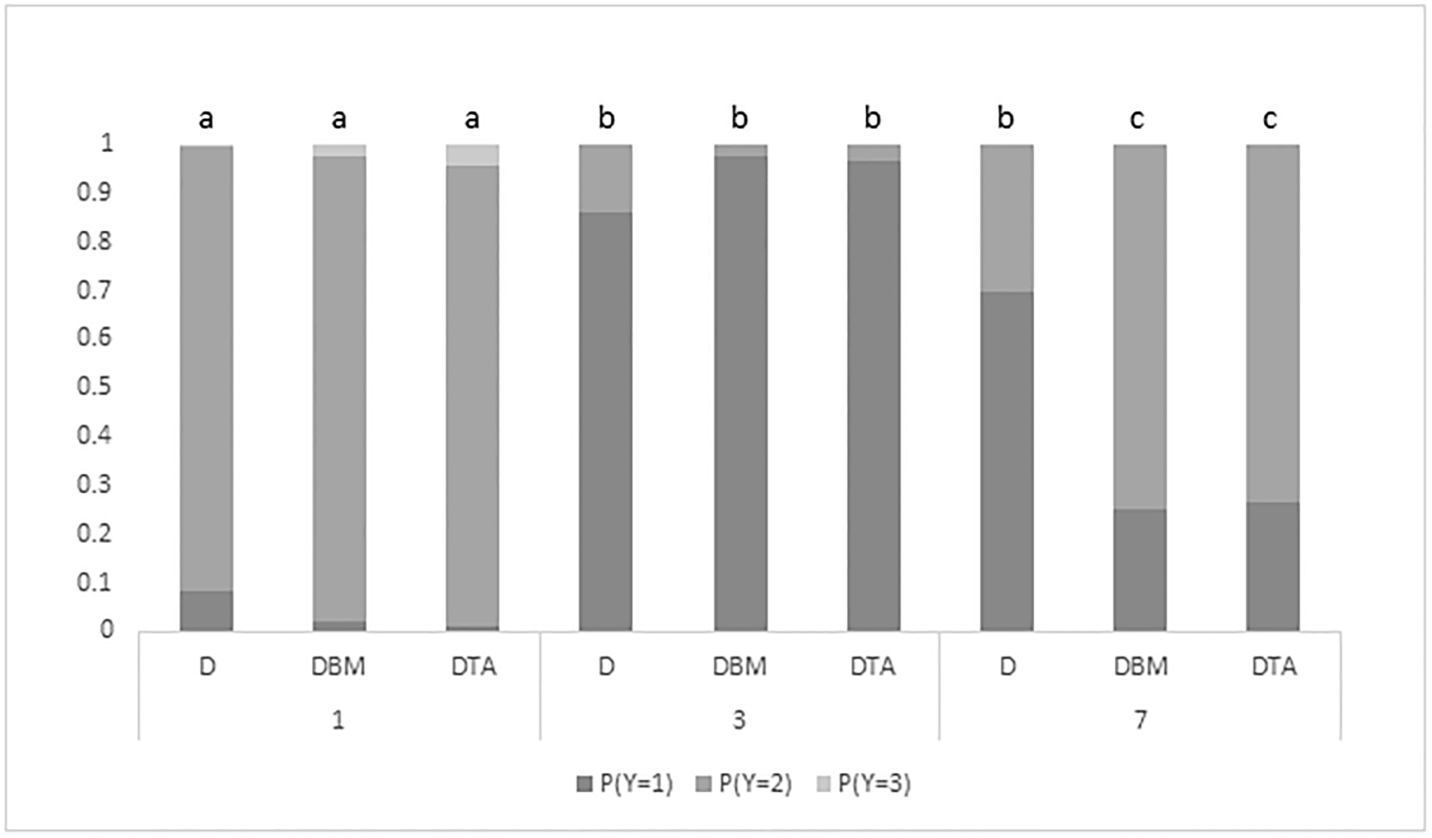
Probability of dehorning wounds from calves in each treatment group displaying morphology scores (Y; 1, 2, 3) on days 1, 3 and 7 following treatment. D = dehorned; DBM = dehorned with pre-operative buccal meloxicam; DTA = dehorned with post-operative topical anaesthetic. ^a, b^ Days with different superscripts differ significantly at *P* ≤ 0.05. There were no significant differences between treatments within each time-point (*P* > 0.05). A significant effect was found (*P* < 0.03).

There was a significant treatment x day interaction (*P* = 0.03). All wound morphology scores decreased from day 1 to day 3. Wound morphology scores of DBM and DTA calves increased from day 3 to day 7 (Fig 3).

## Discussion

The aim of this study was to evaluate the effects of TA and BM on behaviour, maximum wound temperature and wound morphology, as indicators of pain and inflammation following amputation dehorning of calves. Although these products were investigated as their modes of administration are considered practical for on-farm administration, this study did not demonstrate clear findings of the effects of TA or BM on behaviour, maximum wound temperature or wound morphology when administered alone. No conclusions can be made on the analgesic efficacy of these products for dehorning in calves and further research is required.

In our study, calf behaviour was assessed to identify potential pain-related differences between treatment groups. There were differences detected between undehorned and dehorned calves for some behaviours. However, there were no significant effects of TA or BM on these behaviours. Dehorned calves displayed more head shakes at 2 and 3 h following treatment and more combined point behaviours at 2 h following treatment compared to CON calves, suggesting these behaviours were pain-related. It also suggests an escalation in pain over time in dehorned calves, likely due to a progression in inflammation [15]. This aligns with previous studies that have found head shaking to occur more frequently in dehorned than undehorned calves [4, 16–18]. Similarly, combined pain-related behaviours have been shown to occur more frequently in dehorned compared to undehorned calves [17]. Although at 2 h following treatment, CON and DTA calves displayed fewer head turns than DBM calves, as there were no significant differences between CON, DTA and D calves at this point, it is unclear if this is indicative of effective pain relief from the TA. This behavior may be associated with other irritating factors such as the presence of blood from the wound running into the eyes of the calves, or flies on the wound or body. A previous study found that the combination of lignocaine and ketoprofen had a significant effect on lying, grazing or ruminating, tail shaking and ear flicking between control and dehorned calves during the first 4 h following treatment, whereas this was not as evident when lignocaine or ketoprofen were administered alone [5]. This suggests that a combination of TA and BM may have had a greater effect on the number of head shakes or combined point behaviours following dehorning in the current study. A combination of TA and BM was not assessed due to limited animal numbers for inclusion in the study and should be investigated in future research. In the current study, there was little expression of pain-related behaviours overall and many behaviours were seemingly unaffected by treatment. The behavioural results from the current study may have been affected by the calves being unweaned and potentially focused mainly on reuniting with their mothers [19]. Dairy calves, already separated from their mothers, were used in similar previous studies [4, 5] where treatment differences were more detectable. However, there are studies that have also found little or no difference in post-operative behaviour of dehorned and undehorned control animals [20]. Hence the findings of the present study are not unusual.

Increased surface temperature resulting from hoof lesions [21] and mammary gland infections [22] in dairy cattle, ear lesions in lambs [23] and castration in beef calves [24] has previously been detected through the use of infrared thermography. Maximum wound temperature was greater on days 3 and 7 than on day 1, potentially reflecting the progression of inflammation, which is consistent with previous studies on wound temperature following surgical castration [24] and branding [25]. In the initial stage following tissue injury, vasoactive metabolites are released causing vasodilation of arterial vasculature and hyperthermia. Histamine release from mast cells additionally increases vasodilation and vascular permeability to allow inflammatory cells to enter the perivascular space in the vicinity of the wound. Inflammation leads to wound healing, although it persists until bacteria and debris are cleared. It is characterised by an influx of neutrophils, macrophages and lymphocytes [26] and increased cutaneous cell metabolism and blood flow, observed as increased cutaneous temperature [27]. It is possible that the elevated maximum wound temperatures observed on days 3 and 7 could be due to the presence of inflammation associated with flystrike, as noted anecdotally. Neither TA nor BM affected maximum wound temperature on 1, 3 or 7 days following treatment, suggesting that these products may not have had any effect on inflammation at these time-points. This may be expected with TA, although was unexpected with meloxicam as this NSAID compound is known to inhibit production of inflammatory mediators and has a half-life of 19.97 to 43.29 h [15, 28]. An alternative explanation for the lack of a treatment effect may be that infrared thermography is not suitable to detect drug induced changes in dehorning wound inflammatory status. Previous studies have found no differences in wound surface temperature of calves castrated with and without the NSAIDs ketoprofen [24] or flunixin [29]. Flunixin also has been shown to have no effect on surface temperature of hot-iron brands in cattle [30]. Future research should include alternative measures of inflammation, such as analysis of acute phase proteins.

Wound morphology scores have previously been used to assess inflammation and healing associated with dehorning [31], castration [19, 29, 32, 33] and branding [30, 34] in cattle. Results from this study showed a decrease in wound morphology score from day 1 to day 3 for all calves, suggesting a reduction in inflammation and a development in healing. From day 3 to day 7, an increase in wound morphology score was seen for DBM and DTA calves, possibly due to a progression in flystrike severity, as noted anecdotally. It is difficult to say whether this was a causative effect as although it was noted, there was no formal recording of the presence of flystrike or infection for each individual animal. It was noted that there was a sequential substantial increase in the number of flies and maggots on or within wounds and sinuses throughout the study that would likely have confounded normal wound healing results. Topical anaesthesia has been found to improve wound healing 2 and 4 weeks following mulesing in lambs. However, measurement of wound contraction rather than visual scoring was used to assess healing in this study [35]. Buccal meloxicam has been found to worsen wound conditions 4 and 7 days following surgical castration and 7 days following tail docking in lambs, as measured using a visual scoring system [14]. However, flystrike appeared to confound the results in this study, which is probably also the case for the current study.

In this study, there was no effect of TA or BM on behaviour, maximum wound temperature and wound morphology of calves following amputation dehorning. The timing of the study in summer meant that there were many flies present, resulting in irritation of the wound and flystrike. The potential for flystrike to be a subsequent animal welfare issue following dehorning of calves was highlighted in this study and should be addressed through controlled timing of husbandry procedures if possible. The presence of flies and occurrences of flystrike were likely major confounding factors when evaluating behaviour, maximum wound temperature and wound morphology as indications of pain and inflammation following dehorning, with and without TA and BM, in calves. Therefore, further research is needed to draw conclusions on the efficacy of these products for amputation dehorning of calves.

## Supporting information

S1 File Study data. (XLSX)
